# Optical effects of exposing intact human lenses to ultraviolet radiation and visible light

**DOI:** 10.1186/1471-2415-11-41

**Published:** 2011-12-30

**Authors:** Line Kessel, Lars Eskildsen, Jesper Holm Lundeman, Ole Bjarlin Jensen, Michael Larsen

**Affiliations:** 1 Department of Ophthalmology, Glostrup Hospital, Nordre Ringvej 57, DK-2600 Glostrup, Denmark; 2 DTU Fotonik, Department of Photonics Engineering, Technical University of Denmark, Ørsteds Plads 343, DK-2800 Lyngby, Denmark; 3 DTU Fotonik, Department of Photonics Engineering, Technical University of Denmark, Frederiksborgvej 399, DK-4000 Roskilde, Denmark; 4 Faculty of Health Sciences, University of Copenhagen, Blegdamsvej 3, 2200 Copenhagen, Denmark

## Abstract

**Background:**

The human lens is continuously exposed to high levels of light. Ultraviolet radiation is believed to play a causative role in the development of cataract. In vivo, however, the lens is mainly exposed to visible light and the ageing lens absorbs a great part of the short wavelength region of incoming visible light. The aim of the present study was to examine the optical effects on human lenses of short wavelength visible light and ultraviolet radiation.

**Methods:**

Naturally aged human donor lenses were irradiated with UVA (355 nm), violet (400 and 405 nm) and green (532 nm) lasers. The effect of irradiation was evaluated qualitatively by photography and quantitatively by measuring the direct transmission before and after irradiation. Furthermore, the effect of pulsed and continuous laser systems was compared as was the effect of short, intermediate and prolonged exposures.

**Results:**

Irradiation with high intensity lasers caused scattering lesions in the human lenses. These effects were more likely to be seen when using pulsed lasers because of the high pulse intensity. Prolonged irradiation with UVA led to photodarkening whereas no detrimental effects were observed after irradiation with visible light.

**Conclusions:**

Irradiation with visible light does not seem to be harmful to the human lens except if the lens is exposed to laser irradiances that are high enough to warrant thermal protein denaturation that is more readily seen using pulsed laser systems.

## Background

Cataract is a major health problem, accounting for almost 20 millions cases of blindness globally and an even greater number of cases of low vision [1]. Cataract is characterised by increased absorption and scattering of light by the lens of the eye resulting in a decreased transmission of light to the retina. Evaluating the yellow-brownish discolouration of the lens is an important aspect of grading the severity of cataract [2-4]. Lens chromophores are formed by a number of pathways including photochemical modification of tryptophan [5-9] and denaturation with sugars forming advanced glycation end products and cross-links between lens proteins [10-13].

Exposure to ultraviolet radiation in the UVB (280-320 nm) can induce cataract in animal studies [14,15] and epidemiological studies suggest a link between cortical cataract and exposure to ultraviolet radiation [16-19]. In vivo, the lens is relatively well protected from UVB radiation because the shorter wavelengths of the solar spectrum are absorbed by atmospheric ozone, the upper eye lids and to a lower extent the eyebrows shield the eye [20,21] and because UV is absorbed to a large degree in the cornea and the aqueous humour [22,23]. The natural ageing process of the lens leads to increased lens yellowing [24] and the aged lens contains UV-absorbers and chromophores that absorb both ultraviolet radiation [22,25] and with age increasing proportions of violet, blue and to a lesser extent green light too [26]. The part of the solar spectrum that reaches and becomes absorbed by lens is thus dominated by UVA and the short wavelength segment of the visible spectrum. Whereas the detrimental effect of UVB is well documented in the literature, UVA seems less damaging [7,15,27] and may even bleach lens chromophores [28,29]. The effects of irradiation with visible light have not been described in the literature but since the aged human lens absorbs a great proportion of short wavelength visible light it is very relevant to examine this part of the electromagnetic spectrum for potentially hazardous effects. The aim of the present study was to examine and compare the effects of irradiation with ultraviolet radiation and visible light on the optical properties of naturally aged human lenses. This was done by using different wavelengths of irradiation, different exposure times and irradiation levels, and pulsed and continuous wave laser systems. The effect of the irradiation was documented qualitatively by photographs and quantitatively by measuring the direct transmission of white light before and after irradiation.

## Methods

### Biological material

Human donor lenses were kindly provided by Dr Liesbeth Pels and co-workers of the Corneabank NORI, Amsterdam, the Netherlands. Lenses were procured within 24 hours post mortem and kept at 5°Celsius in minimal essential medium (MEM) until they were used for the experiments (> 4 days post mortem). All lenses were of good optical quality indicating that no swelling had taken place. A few of the lenses had localised opacities of the capsule induced by the postmortem storage. To avoid potential problems with scattering from these localised opacites the lens capsule was gently removed before irradiation and the lens placed between two glass mounting plates kept apart by an adjustable spacer. Removing the capsule did not influence the way a lens responded to irradiation as judged by comparing the two lenses from the same donor in a control experiment including 2 sets of lenses.

Only non-identifiable donor material was used. The study adhered to the tenets of the Helsinki Declaration and it was approved by the medical ethics committee of Copenhagen County.

### Evaluation of the effects of irradiation

The effect of irradiation was evaluated by direct white light transmission measurements, visual inspection and by photography using a Canon EOS 30D digital camera equipped with a Canon compact-macro lens. The transmission of white light was measured using a broad band white light source (DT-Mini-2-GS, Micropack, Ocean Optics, the Netherlands) that was fibre coupled to a collimating set of lenses in front of the human lens. After passing through the mounted human lens, transmitted light was detected by focusing outcoming light into an optical fibre that was connected to an Avantes Spectrometer (AvaSpec-2048-2, Avantes BV, The Netherlands). The resolution of the spectrometer was 0.3 nm. Direct lens transmission was calculated as the ratio between the intensity of emitted (*I_lens_*) and incident (*I_incident_*) light after correction for background levels (*I_dark_*) of light (Eq. 1):

(1) $$T=\frac{I_{lens}-I_{dark}}{I_{incident}-I_{dark}}$$

Transmission spectra were normalized to a nominal transmission of 100% between 600 and 700 nm.

### Laser systems

Four different laser systems were examined: a pulsed nanosecond laser at 355 nm (third harmonic Nd:YAG, pulse duration 4.2 ns, repetition rate 13 kHz), a pulsed femtosecond laser at 400 nm (frequency double Ti:Sapphire (Mira 900, Coherent, USA) after amplification by a regenerative amplifier (RegA, Coherent, USA), pulse duration 150-250 fs (10^-15 ^seconds), repetition rate 275 kHz), a continuous wave (cw) frequency doubled diode laser at 405 nm [30], and a cw frequency doubled solid state Nd:YAG laser at 532 nm (LSR532U-200, Lasever, China). Laser energy output was measured using a thermopile detector and was adjusted to the desired irradiance using a graded neutral density filter. Irradiances are reported as the radiant power per area (W/cm2) according to CIE standards [31]. The lenses were irradiated with a collimated laser beam. The area of interest was defined by fixing a circular aperture (1.4 mm in diameter) on the front surface of the lens mounting system. The laser beam cross-section was kept larger than the aperture to ensure that the entire area of the aperture was irradiated. All transmission measurements were performed through the aperture.

## Results

### Pulsed ultraviolet radiation at 355 nm short and long exposures

**Figure 1:**
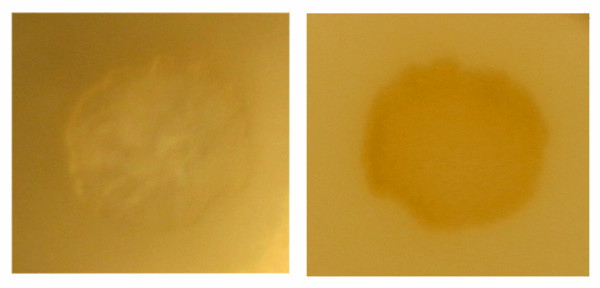
**Photowhitening and photodarkening**. Close-up photographs showing the white (left) and brown (right) lesions produced by irradiation with a 355 nm pulsed laser. The lesions are circular with a diameter of 1.4 mm, corresponding to the aperture used.

**Figure 2:**
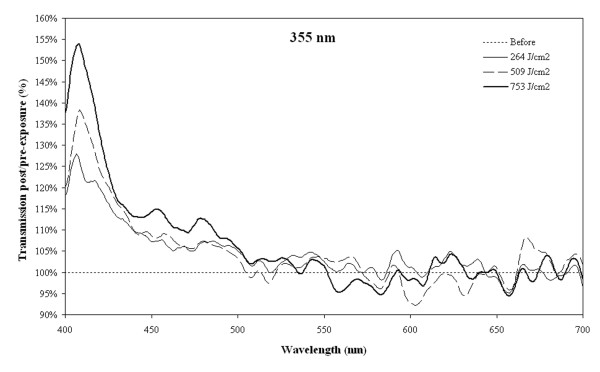
**Transmission changes after 355 nm**. Changes in transmission after irradiation of a 72 year old human donor lens with a 355 nm pulsed laser. Each graph represents a 60 minute exposure duration. This was the only lens that showed a dose-response photobleaching after exposure to 355 nm.

Six lenses from donors aged 54 to 72 years were irradiated at 355 nm and were found to develop white lesions instantaneously at a mean laser irradiance of 65 mW/cm^2 ^(corresponding to a pulse energy of 0.4 μJ) or higher, Figure 1. White lesions were avoided when laser irradiance was reduced to 16 mW/cm2 (corresponding to a pulse energy of 0.1 μJ) but prolonged exposure (~72 hours) led to brown lesions, Figure 1. In all 6 lenses transmission at 355 nm was < 1% before exposure. No light transmission was detectable through the white or brown lesions. In a single case a dose-dependent photobleaching was observed at a laser irradiance of 75 mW/cm^2 ^that produced photodamage in all other lenses, see Figure 2. In this single case of photobleaching, a dose-dependent increase in transmission of short wavelengths was noted with a maximum effect around 410 nm.

### Pulsed and continuous wave violet light at 400 and 405 nm short, intermediate and long exposures

**Figure 3:**
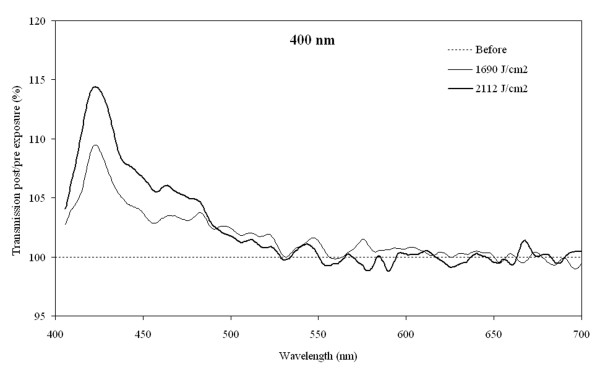
**Transmission changes after 400 nm**. Changes in transmission as a function of wavelength after irradiation of a 64 year old human donor lens with a 400 nm femtosecond pulsed laser for 18 h 47 minutes and 23 h and 28 minutes. The transmission before irradiation was set to 100% for all wavelengths.

The effects of pulsed and continuous wave (cw) lasers were compared using a cw (405 nm) and a femtosecond pulsed laser (400 nm). Seven lenses from donors aged 64 to 73 years were irradiated with the pulsed laser and 7 lenses from donors aged 57 to 75 years were irradiated with the cw system. Light transmission before exposure was on average 1.1% at 400 nm and it was 2.2% at 405 nm. Using the pulsed laser system, white lesions were produced instantaneously with pulse energy densities < 0.4 μJ/cm^2 ^or higher. No light transmission was detectable through the white lesions. Lower laser irradiances resulted in macroscopically visible photobleaching and increased transmission of predominantly blue light, Figure 3. Blue light transmission from 450-490 nm increased by 4.7 - 18.8% after irradiation. A similar result was obtained for the cw irradiation with production of white lesions at laser irradiances < 165 mW/cm^2 ^while lower irradiances led to photobleaching. Blue light transmission from 450-490 nm increased by 9.7 to 34.2% after irradiation. Brown lesions were not observed after irradiation with the violet lasers (exposure durations up to 18 hours).

### Continuous wave green light at 532 nm short and intermediate exposures

**Figure 4:**
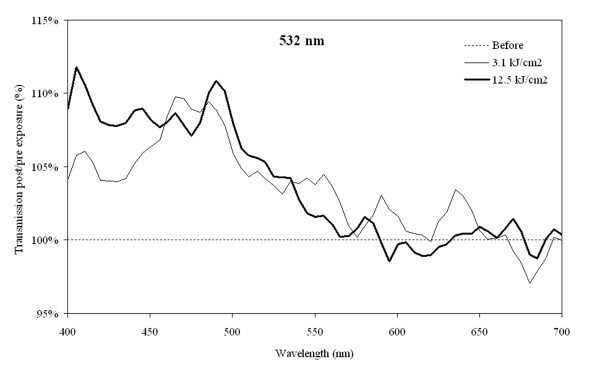
**Transmission changes after 532 nm**. Changes in transmission of a human donor lens (aged 72 years) during irradiance with a 532 nm continuous wave laser for 10 and 40 minutes. Transmission before irradiation was set to 100% for all wavelengths.

All three lenses (aged 68 to 72 years) irradiated with a green cw laser had a light transmission at 532 nm of 57- 84% before irradiation and they consistently showed a minor increase in transmission of 8.0 - 8.3% in the blue region from 450-490 nm after irradiation by ~1.6 kJ/cm^2^, Figure 4. The effects were barely visible macroscopically. Increasing the radiation dose up to 105 minutes (~3 kJ/cm^2^) did not result in further photobleaching or formation of white or brown lesions.

## Discussion

The main purpose of the present work was to examine and compare the effect of ultraviolet and short wavelength visible irradiation on naturally aged human lenses. We found that irradiation with short wavelengths either led to photobleaching or photodamage seen as whitish lesion formed instantaneously upon exposure to very high laser irradiances of UVA or violet lasers or dark lesions that were seen only after prolonged exposure to UVA.

The present study has some limitations. For 355 nm we only had access to a pulsed laser system and at 532 nm we only had access to a continous wave laser system. For this reason we compared the effect of pulsed and cw lasers around 400 nm and we found that pulsed lasers are very likely to produce photodamage because of their high pulse energy density. Due to the differences in laser system is was necessary to use very long exposure times in some experiments to get below the pulse energy density where photo-whitening was observed. Potentially this could have influenced the results. However, based on the experiments around 400 nm we observed the same response to irradiation when the lenses had been exposed to a total of 1.6 kJ/cm2 during a time period of 18 hours for the femtosecond experiments or 15 minutes for the cw experiments. Based on these few observations it seems likely that the photobleaching is independent on the exposure time but dependent on the exposure dose. The number of wavelengths studied and the number of lenses studied for each wavelength and type of irradiation (pulsed versus cw) are small and this means that the given values for photodamage cannot be interpreted directly as threshold levels.

UVA absorption has previously been shown to increase the temperature of the lens [32] and thus the white immediate lesions are likely to be thermal, induced by absorption and dissipation of heat in the vicinity of the absorbing chromophores. Lens proteins are prone to aggregation by heating [33] and since lens transparency is intimately related to the three-dimensional arrangement of the lens proteins [34,35] protein coagulation and aggregation will increase optical density [36]. Our results may need to be taken into consideration when interpreting previous findings of lens protein aggregation in studies using pulsed UV irradiation [36-39]. The average human exposure to ambient ultraviolet radiation is around 25 kJ/m^2^/year (in the USA) [40] and the ANSI standard maximum permissible exposure limit for photochemical damage for a point source laser beam at 355 nm is 1 J/cm^2 ^[41]. The white lesions were only produced with high laser irradiances that a living human would not experience unless accidentally exposed to very strong laser sources.

Photodarkening was only noted after very high doses of UVA (< 4 kJ/cm^2^). The nature of the photochemical reactions leading to the observed photodarkening is not known. A large number of chromophores have been identified in the aged human lens. Photooxidation is believed to play a role for cataractogenesis [42] and was most likely also involved in the photodarkening we observed.

Photobleaching was observed upon exposure to the violet and green lasers. It was observed only once after UVA irradiation using doses that in all other cases lead to photodamage. It is not known why that single lens was bleached and not damaged since the optical properties of the lens did not differ substantially from the other lenses although it was the oldest, and hence the most darkly coloured, of the lenses studied at 355 nm. The phenomenon of photobleaching was previously reported after exposure to UVA [28,29]. It was not associated with any signs of opacification of the lens during an observational period of one week after exposure but the long term effects are unknown. The bleaching was localized and remained stable for one week after exposure, showing that the chromophores that were bleached are not diffusible. The biochemical processes behind the photobleaching are unknown and were not assessed in the present study. We analyzed the transmission properties of the lenses before and after exposure and found a decreased absorption of short wavelengths after irradiation in the lenses that were photobleached. The age-induced increased absorption of short wavelengths by the lens is well-known [23,26] and different models characterising the age-induced spectral changes have been presented [43,44]. The observed photobleaching was a combination of decreased absorption by the young and old lens chromophores in the van de Kraats model [44].

## Conclusions

In conclusion, the present study showed that UVA, violet and green light seems to be optically harmless to the human lens except for prolonged exposure to high dose UVA or short term exposure to very intense laser radiation capable of inducing thermal damage. Notably, pulsed lasers more readily produce thermal damage due to the high pulse energy densities and should only be used with precaution in future research.

## Competing interests

The authors declare that they have no competing interests.

## Authors' contributions

LK conceived the study, carried out the experiments and drafted the manuscript. LE, JHL and OBJ carried out the experiments. ML contributed to conceiving the experiments. All authors critically read and approved the final manuscript.

## Pre-publication history

The pre-publication history for this paper can be accessed here:

http://www.biomedcentral.com/1471-2415/11/41/prepub

## References

[B1] Javitt JC Wang F West SK Blindness due to cataract: Epidemiology and prevention Annual Review of Public Health 1996 17 159 177 10.1146/annurev.pu.17.050196.001111 8724222

[B2] Pirie A Color and solubility of the proteins of human cataracts Invest Ophthalmol 1968 7 634 650 5727811

[B3] Chylack LT Jr Wolfe JK Singer DM Leske MC Bullimore MA Bailey IL The Lens Opacities Classification System III. The Longitudinal Study of Cataract Study Group Arch Ophthalmol 1993 111 831 836 10.1001/archopht.1993.01090060119035 8512486

[B4] The age-related eye disease study (AREDS) system for classifying cataracts from photographs: AREDS report no. 4 Am J Ophthalmol 2001 131 167 175 11228291 10.1016/s0002-9394(00)00732-7 PMC2032014

[B5] Roberts JE Finley EL Patat SA Schey KL Photooxidation of lens proteins with xanthurenic acid: a putative chromophore for cataractogenesis Photochem Photobiol 2001 74 740 744 10.1562/0031-8655(2001)074<0740:POLPWX>2.0.CO;2 11723804

[B6] Aquilina JA Carver JA Truscott RJ Oxidation products of 3-hydroxykynurenine bind to lens proteins: relevance for nuclear cataract Exp Eye Res 1997 64 727 735 10.1006/exer.1996.0258 9245903

[B7] Mizdrak J Hains PG Truscott RJ Jamie JF Davies MJ Tryptophan-derived ultraviolet filter compounds covalently bound to lens proteins are photosensitizers of oxidative damage Free Radic Biol Med 2008 44 1108 1119 10.1016/j.freeradbiomed.2007.12.003 18206985

[B8] Truscott RJ Age-related nuclear cataract-oxidation is the key Exp Eye Res 2005 80 709 725 10.1016/j.exer.2004.12.007 15862178

[B9] Pirie A Formation of N'-formylkynurenine in proteins from lens and other sources by exposure to sunlight Biochem J 1971 125 203 208 5168391 10.1042/bj1250203 PMC1178040

[B10] Monnier VM Cerami A Nonenzymatic browning in vivo: possible process for aging of long-lived proteins Science 1981 211 491 493 10.1126/science.6779377 6779377

[B11] Ulrich P Cerami A Protein glycation, diabetes, and aging Recent Prog Horm Res 2001 56 1 21 10.1210/rp.56.1.1 11237208

[B12] Chellan P Nagaraj RH Early glycation products produce pentosidine cross-links on native proteins. novel mechanism of pentosidine formation and propagation of glycation J Biol Chem 2001 276 3895 3903 10.1074/jbc.M008626200 11076948

[B13] Padayatti PS Ng AS Uchida K Glomb MA Nagaraj RH Argpyrimidine, a blue fluorophore in human lens proteins: high levels in brunescent cataractous lenses Invest Ophthalmol Vis Sci 2001 42 1299 1304 11328743

[B14] Mody VC Jr Kakar M Elfving A Soderberg PG Lofgren S Ultraviolet radiation-B-induced cataract in albino rats: maximum tolerable dose and ascorbate consumption Acta Ophthalmol Scand 2006 84 390 395 10.1111/j.1600-0420.2006.00640.x 16704705

[B15] Oriowo OM Cullen AP Chou BR Sivak JG Action spectrum and recovery for in vitro UV-induced cataract using whole lenses Invest Ophthalmol Vis Sci 2001 42 2596 2602 11581205

[B16] WHO Fact sheet N 305 Global disease burden from solar ultraviolet radiation 2006

[B17] Taylor HR Ultraviolet radiation and the eye: an epidemiologic study Trans Am Ophthalmol Soc 1989 87 802 853 2562534 PMC1298564

[B18] McCarty CA Taylor HR A review of the epidemiologic evidence linking ultraviolet radiation and cataracts Dev Ophthalmol 2002 35 21 31 12061276 10.1159/000060807

[B19] West SK Longstreth JD Munoz BE Pitcher HM Duncan DD Model of risk of cortical cataract in the US population with exposure to increased ultraviolet radiation due to stratospheric ozone depletion Am J Epidemiol 2005 162 1080 1088 10.1093/aje/kwi329 16251390

[B20] Sliney DH Exposure geometry and spectral environment determine photobiological effects on the human eye Photochem Photobiol 2005 81 483 489 10.1562/2005-02-14-RA-439.1 15755194

[B21] Sliney DH Geometrical gradients in the distribution of temperature and absorbed ultraviolet radiation in ocular tissues Dev Ophthalmol 2002 35 40 59 12061278 10.1159/000060809

[B22] Boettner EA Wolter JR Transmission of the ocular media Invest Ophthalmol Vis Sci 1962 1 776 783

[B23] Ambach W Blumthaler M Schopf T Ambach E Katzgraber F Daxecker F Spectral transmission of the optical media of the human eye with respect to keratitis and cataract formation Doc Ophthalmol 1994 88 165 173 10.1007/BF01204614 7781484

[B24] Lerman S Borkman R Spectroscopic evaluation and classification of the normal, aging, and cataractous lens Ophthalmic Res 1976 8 335 353 10.1159/000264841

[B25] Weale RA Age and the transmittance of the human crystalline lens J Physiol 1988 395 577 587 3411488 10.1113/jphysiol.1988.sp016935 PMC1192010

[B26] Kessel L Lundeman JH Herbst K Andersen TV Larsen M Age-related changes in the transmission properties of the human lens and their relevance to circadian entrainment Journal of Cataract & Refractive Surgery 2010 36 308 312 10.1016/j.jcrs.2009.08.035 20152615

[B27] Ortwerth BJ Bhattacharyya J Shipova E Tryptophan metabolites from young human lenses and the photooxidation of ascorbic acid by UVA light Invest Ophthalmol Vis Sci 2009 50 3311 3319 10.1167/iovs.08-2927 19264899

[B28] Ortwerth BJ Chemoganskiy V Olesen PR Studies on Singlet Oxygen Formation and UVA Light-mediated Photobleaching of the Yellow Chromophores in Human Lenses Exp Eye Res 2002 74 217 229 10.1006/exer.2001.1114 11950232

[B29] Kessel L Kalinin S Soroka V Larsen M Johansson LBA Impact of UVR-A on whole human lenses, supernatants of buffered human lens homogenates, and purified argpyrimidine and 3-OH-kynurenine Acta Ophthalmologica Scandinavica 2005 83 221 227 10.1111/j.1600-0420.2005.00388.x 15799737

[B30] Lundeman JH Jensen OB Andersen PE ndersson-Engels S Sumpf B Erbert G High power 404 nm source based on second harmonic generation in PPKTP of a tapered external feedback diode laser Optics Express 2008 16 2486 2493 10.1364/OE.16.002486 18542327

[B31] Sliney DH Radiometric quantities and units used in photobiology and photochemistry: recommendations of the Commission Internationale de L'Eclairage (International Commission on Illumination) Photochem Photobiol 2007 83 425 432 10.1562/2006-11-14-RA-1081 17115802

[B32] Okuno T Kojima M Hata I Sliney DH Temperature rises in the crystalline lens from focal irradiation Health Phys 2005 88 214 222 10.1097/01.HP.0000146581.72675.aa 15706141

[B33] Regini JW Grossmann JG Timmins P Harding JJ Quantock AJ Hodson SA X-ray- and neutron-scattering studies of alpha-crystallin and evidence that the target protein sits in the fenestrations of the alpha-crystallin shell Investigative Ophthalmology & Visual Science 2007 48 2695 2700 10.1167/iovs.06-0559 17525201

[B34] Stradner A Foffi G Dorsaz N Thurston G Schurtenberger P New insight into cataract formation: Enhanced stability through mutual attraction Physical Review Letters 2007 99 10.1103/PhysRevLett.99.198103 18233120

[B35] Takemoto L Sorensen CM Protein-protein interactions and lens transparency Exp Eye Res 2008 87 496 501 10.1016/j.exer.2008.08.018 18835387 PMC2666974

[B36] Dillon J Roy D Spector A Walker ML Hibbard LB Borkman RF UV laser photodamage to whole lenses Exp Eye Res 1989 49 959 966 10.1016/S0014-4835(89)80019-3 2612589

[B37] Ostrovsky MA Sergeev YV Atkinson DB Soustov LV Hejtmancik JF Comparison of ultraviolet induced photo-kinetics for lens-derived and recombinant beta-crystallins Mol Vis 2002 8 72 78 11951082

[B38] Hott JL Borkman RF Concentration-Dependence of Transmission Losses in Uv-Laser Irradiated Bovine Alpha-Crystallin, Beta-H-Crystallin, Beta-L-Crystallin and Gamma-Crystallin Solutions Photochemistry and Photobiology 1993 57 312 317 10.1111/j.1751-1097.1993.tb02293.x 8451296

[B39] Li DY Borkman RF Photodamage to calf lenses in vitro by excimer laser radiation at 308, 337, and 350 nm Invest Ophthalmol Vis Sci 1990 31 2180 2184 2211014

[B40] Godar DE Wengraitis SP Shreffler J Sliney DH UV doses of Americans Photochem Photobiol 2001 73 621 629 10.1562/0031-8655(2001)073<0621:UDOA>2.0.CO;2 11421067

[B41] American National Standard for Safe Use of Lasers, ANSI Z136.1-2007 2007 Orlando, Fl 32826:Laser Institute of America

[B42] Davies MJ Truscott RJ Photo-oxidation of proteins and its role in cataractogenesis J Photochem Photobiol B 2001 63 114 125 10.1016/S1011-1344(01)00208-1 11684458

[B43] Pokorny J Smith VC Lutze M Aging of the human lens Appl Opt 1987 26 1437 1440 10.1364/AO.26.001437 20454339

[B44] van de Kraats J van ND Optical density of the aging human ocular media in the visible and the UV J Opt Soc Am A Opt Image Sci Vis 2007 24 1842 1857 10.1364/JOSAA.24.001842 17728807

